# The Binding of PD-L1 and Akt Facilitates Glioma Cell Invasion Upon Starvation via Akt/Autophagy/F-Actin Signaling

**DOI:** 10.3389/fonc.2019.01347

**Published:** 2019-12-03

**Authors:** Ruo Qiao Chen, Xiao Hong Xu, Feng Liu, Chun Yang Li, Yuan Jun Li, Xiang Rui Li, Guo Yong Jiang, Feng Hu, Di Liu, Feng Pan, Xin Yao Qiu, Xiao Qian Chen

**Affiliations:** ^1^School of Basic Medicine, Tongji Medical College, Huazhong University of Science and Technology, Wuhan, China; ^2^Department of Pathophysiology, School of Basic Medicine, Tongji Medical College, Huazhong University of Science and Technology, Wuhan, China; ^3^Department of Neurosurgery, Tongji Hospital, Tongji Medical College, Huazhong University of Science and Technology, Wuhan, China; ^4^Department of Urology, Union Hospital, Tongji Medical College, Huazhong University of Science and Technology, Wuhan, China

**Keywords:** glioblastoma multiforme, CD274, p62, autophagic influx, ischemia

## Abstract

Glioma, especially glioblastoma, is pathologically characterized by high aggressiveness, which largely contributed to the ineffectiveness of current therapies. It has been recently reported that intrinsic PD-L1 can regulate tumor malignancy, whereas underlying mechanisms remain mostly unclear. Here, we report a novel mechanism by which PD-L1 promotes glioma cell infiltration. In orthotopic glioma models, PD-L1 expression was up-regulated predominantly in glioma cells in the infiltrating front. For PD-L1-overexpressed glioma cells, PI3K/Akt and actin regulations were among the top six most altered signaling pathways as detected by RNA-sequencing. PD-L1 significantly activated Akt/F-actin signaling while suppressed autophagic signaling upon cell starvation. Mechanistically, PD-L1 preferentially bound to Akt among various PI3K/Akt signaling proteins. Serial truncation identified the interaction between the 128-237aa fragment of PD-L1 and the 112-480aa fragment of Akt, which facilitates the membrane translocation/activation of Akt, and was unaffected by Perifosin (specific p-Akt inhibitor targeting Akt PH-domain). Taken together, our data indicate that in glioma cells, PD-L1 is induced to prevent autophagic cytoskeleton collapse via Akt binding/activation, facilitating glioma cell invasion upon starvation stress.

## Introduction

Glioma, the most common primary intracranial tumor, originates from various types of cells. The most malignant glioma is glioblastoma, or glioblastoma multiforme (GBM), which accounts for around 21% of glioma and can be primary or secondary from grade II-III glioma. Recently, glioma is also classified into three types based on its molecular markers: 1p/19q deletion, IDH mutation and TERT promoter mutation. Among them, grade IV glioma mainly has the TERT promoter mutation accompanied by EGFR/PTEN mutation. Current treatments for glioma remain conventional surgery, radiotherapy and chemotherapy. However, glioma is extremely easy to relapse after treatment, with an average survival time of only 14 months for GBM patients after treatment (1). It is urgent to find new and effective therapies in order to prolong the survival time of GBM patients.

The pathological features of GBM are necrosis, high invasiveness and microvascular hyperplasia (2), which are closely related to abnormal energy metabolism. It is generally considered that solid malignant tumors including glioma suffer ischemia/hypoxia throughout their growth. For example, it is estimated that glioma tissues have wide but heterogeneous hypoxia with oxygen concentrations ranging from 0.1 to 2.5% (3). Clinical data reveal that severer GBM necrosis closely correlates to faster GBM progress and shorter patient survival time (1, 2). Upon energy stress such as ischemia, autophagy is a major mechanism to maintain cellular energy homeostasis, which is highly regulated by conserved autophagic influx signaling involving PI3K/Akt/mTOR, p62, Beclin-1 and LC3 (4–6). Higher activity levels of autophagy are widely detected in glioma tissues, particularly around necrotic tissues (7). It is reported that inducing autophagy can either improve the efficacy of chemotherapy (8) or induce chemotherapy-resistance in glioma (9). Large-scale gene knockout screening shows that mitochondrial energy-metabolism genes are necessary for anoxic adaptive growth of a variety of tumors, including GBM (10). Specifically, Kim et al. (11) reported in recent research an increase of mitochondrial SHMT2 (serine hydroxymethyltransferase) in ischemic necrosis tissues of GBM patients, this enhances the anaerobic energy metabolism of GBM cells and promotes survival of GBM cells under ischemic environment. The mechanisms by which ischemia stimulates GBM development are extremely complicated and far from clear.

Programmed cell death ligand-1 (PD-L1, i.e., CD274 or B7H1) is a key negative regulator for immune inhibitory axis signaling controlling T-lymphocyte infiltration inside solid tumors. Previous studies reported that PD-L1 is widely expressed in glioma cell lines (12, 13) and most human glioma specimens (14). However, data of PD-L1 levels and subcellular distributions in human glioma tissues vary greatly (15). Most studies reported that higher PD-L1 expression was correlated with higher glioma grades (16–18) and worse prognosis (17, 19–22), while opposite results were also reported (14, 17, 23). PTEN mutation/deletion (in 36% of glioma) is closely associated with higher PD-L1 expression in glioma (24). Present evidence suggests that PI3K/Akt is a major pathway controlling PD-L1 expression in cancer cells. For mTOR, a key downstream signaling molecule in PI3K/Akt pathway, mTOR complex 1 (mTORC1) mainly mediates PI3K/Akt-induced cell autophagy (25) and mTORC2 mediates Akt-induced cell survival (26).

PD-L1 is recently considered to be oncogenic. PD-L1 knockdown significantly decreases tumor volume in murine ovarian cancer, melanoma (27), murine medulloblastoma (28), and U87 glioma in nude mice (29), while PD-L1 overexpression promotes glioma development (29). Mechanistically, PD-L1 regulates cell growth, proliferation, apoptosis, autophagy, migration and invasion in various cancers via modulating PI3K/Akt/mTOR and Ras/Erk/EMT signalings (27, 29–31). Further exploration of biological roles and mechanisms of PD-L1 in glioma will provide therapeutic cues including targeted immunotherapies for glioma.

In the present study, we first reported that PD-L1 was mostly prominent in those highly aggressive infiltrating glioma cells *in vivo*. Our data revealed that PD-L1 promoted glioma cell infiltration via starvation-induced Akt/autophagy/F-actin signaling. Particularly, we dissected PD-L1-Akt binding fragments and elucidated how PD-L1/Akt interactions activated its downstream cascades.

## Materials and Methods

### Drugs and Antibodies

Cloroquine (CQ, Selleck Chemicals, Shanghai, China) was used at a final concentration of 25 μM. LY294002 (PI3K inhibitor, Cell Signaling Technology, MA, USA) was used at a final concentration of 0–50 μM. Perifosine (inhibitor of Akt PH-domain, Selleck Chemicals, Shanghai, China) was used at a final concentration of 0–25 μM. Primary antibodies against phospho-Akt (Ser473, #D9E), total Akt (#9272), phospho-mTOR, total mTOR,pP62, phospho-P70S6K, LC3B were purchased from Cell Signaling Technology (MA, USA). Antibodies against PD-L1/CD274 (PA5-28115, Thermo Fisher, IL, USA), PD-L1 (ABM4E54, Abcam, Cambridge, UK), GST (Z-5, Santa Cruz Biotechnology, TX, USA), Beclin1 (Santa Cruz Biotechnology, TX, USA), N-terminal GFP (Sigma), β-actin (20536–1-AP, Proteintech, Wuhan, China), Na^+^/K^+^ ATPase α1 (Proteintech, Wuhan, China), β-tubulin (Proteintech, Wuhan, China) were commercially purchased.

### Plasmids and Transfection

GFP-LC3 plasmid was a gift from Dr. He Li (Huazhong University of Science and Technology). GST-kRas/PTEN/Akt1/Akt2/Akt3/EGFR VIII/PKA plasmids were gifts from Dr. Haian Fu (Emory University). PD-L1-EGFP expressing-plasmid was purchased from GeneChem (Shanghai, China). Full-length and truncated human PD-L1 coding cDNA was PCR amplified from PD-L1-EGFP plasmids and cloned into NV (N-terminal Venus 1-157aa) vector including NV-PD-L1 FL (full length 1-290 aa), NV-PD-L1-T1-259 (truncate 1-259aa), T128-259, T128-239, T1-178. PD-L1 cloned into pDEST-26 or p-FU-Venus vector was used to express GST-PD-L1 and Venus-PD-L1 FL1-290 or T19-290. Full-length and truncated human Akt1 cDNA was PCR amplified from GST-Akt1 plasmid and cloned into p-FU-Venus vector for Venus-Akt1, Akt1-T1-111, and Akt1-T122-480. Akt1-Y176A and Akt1-K14R mutants were constructed by using overlapping-PCR from p-FU-Venus-Akt1 plasmid. Transfection of plasmids was performed by using Lipofectamine 2000 (11668, Life Technologies, CA, USA) according to the manufacturer's instructions.

### RNA-Sequencing and Enrichment Analysis

Three 100-mm dishes of U251/PD-L1 and U251/Vec stable cell lines were subjected to total mRNA isolation, cDNA libraries construction and sequencing at Illumina HiSeq sequence platform (PE150) with 6G clean data by Novogene Bioinformatics Institute (Shanghai, China). Differential gene expression analysis between U251/PD-L1 and U251/Vec groups was performed using the DESeq2 R package (1.10.1). The resulting *P*-values were adjusted using the Benjamini and Hochberg's approach for controlling the false discovery rate. Differently expressed genes (DEGs) with *P* < 0.005, adjusted *P* < 0.05 (Padj) and absolute changing fold ≥1.2 were subjected to Kyoto Encyclopedia of Genes and Genomes (KEGG) pathway and Gene Ontology (GO) enrichment analysis. KEGG pathways and GO terms with Padj < 0.05 were considered significantly enriched by DEGs. We have submitted our data to NCBI (https://www.ncbi.nlm.nih.gov/geo/query/acc.cgi?acc=GSE107581).

### Orthotopic Mouse Glioma Model

All animal handling and experiments were performed in accordance with NIH guidelines and approved by the Ethics Committees of Huazhong University of Science and Technology. The mice were group housed in the Animal Core Facility of Tongji Medical College under a 12 h light/dark cycle with *ad libitum* access to food and water. Briefly, adult Kunming male mice (18–20 g) were anesthetized with chloral hydrate (350 mg/kg) and a burr hole was drilled in the skull 0.5 mm posterior to the bregma and 2.0 mm lateral to the midline. A 10-μl Hamilton syringe (26 gauge, Reno, NV) containing 20,000 G422 cells (mouse GBM cell line) in 1 μl of PBS was advanced to a depth of 3.5 mm from the skull surface and then withdrawal 0.3 mm. Cell suspension was delivered at the rate of 1 μl/min. After cell implantation, the needle was left in place for 6 min before withdrawal. After 6–14 d of cell inoculation, the mice were perfused with 4% paraformaldehyde (PFA) and the brains were paraffin-embedded.

### Hematoxylin-Eosin (HE) Staining and Immunohistochemistry (IHC)

IHC was performed as previously reported (29). Paraffin-embedded mouse brain tissues (bearing tumor) were cut into 4 μm-thick slices for H&E staining and IHC analysis. Briefly, the slices were deparaffinized in xylene and antigen-retrieved by microwave processing. After 1 h of blocking with 5% bovine serum albumin in PBS, the slices were incubated with primary antibodies (PD-L1, Abcam, UK) overnight at 4°C, followed by corresponding secondary antibody incubation (Polink-1 HRP DAB Detection System, ZSGB-BIO, China). The immunoreaction was visualized with diaminobenzidine tetrachloride. The brain images were scanned with an automatic slice scanning system-SV120 (Olympus, Tokyo, Japan). The tumor parenchyma rim was delineated with black dashed ellipse circle, while the infiltration frontiers was delineated with blue or white dashed irregular circle.

### Cell Culture and Starvation

Human glioblastoma cell lines U251, LN229, and human embryonic kidney 293T cell line were purchased from American Tissue Culture Collection (MA, USA) or China Center for Type Culture Collection (Wuhan, China). U251, LN228, U87MG with stable PD-L1 overexpression (U251/vec or PD-L1, LN229/vec or PD-L1) or knockout (U251/sgGFP or sgPD-L1) were generated as previously described (29). All cell lines were cultured in Dulbecco's modified Eagle's medium (DMEM, Gibco, CA, USA) supplemented with 10% FBS (Gemini, CA, USA) and 1% Penicillin-Streptomycin Solution (Hyclone, Thermo, Beijing, China). Fresh Earle's balanced salt solution (EBSS, GIBCO BRL, USA) media was used to induce cell starvation at 24 h after transient transfection or initial seeding. Cells were washed with EBSS media for three times and then incubated with EBSS media for various time points.

### Western Blotting Analysis

Western blotting analysis was performed as previously reported (29). Briefly, the cell lysates were collected and dispersed in radio-immunoprecipitation assay lysis buffer containing phenylmethane-sulfonyl fluoride. Equal amounts of total proteins were subjected to sodium dodecyl sulfate polyacrylamide gel electrophoresis and electro-transferred onto nitrocellulose filter membranes (Merck Mil- lipore, Cork, Ireland). The blots were incubated with corresponding primary and secondary IRDye 800 or IRDye 680 CW-conjugated goat anti-rabbit or anti-mouse IgG antibodies (LI-COR Biosciences, Lincoln, USA). The labeled bands were visualized and quantified by Odyssey Infrared Imaging System (LI-COR Biosciences, MA, USA).

### Palloidin Staining and Immunofluorescence

Immunofluorescence staining was performed as previously reported (29). Cells in 35-mm culture dishes were fixed, permeabilized, blocked, and then incubated with primary and corresponding Dylight 488-labeled secondary antibodies (Abbkine, CA, USA). F-actin was stained with rhodamine-phalloidin (Yisheng Bioengineering Institute, China) according to the manufacturer's instructions. Hoechst 33342 was used to stain the nucleus. For paraffin-embedded tissues, rat brain slices were deparaffinized, rehydrated, antigen unmasked, blocked with 5% bovine serum albumin (BSA) and then incubated with primary antibodies and corresponding Dylight 488/594- labeled secondary antibodies. Micrographs were taken under the same conditions with a conventional fluorescent microscope (Olympus, Tokyo, Japan).

### GFP-LC3 Punctate Quantification

U251/sgGFP or U251/sgPD-L1 cells were transiently transfected with pGFP-LC3 plasmids for 24 h. Then, the cultures were subjected to EBSS treatment for 12 h and fixed. U251 cells expressing GFP-LC3 were randomly photographed under 400×-magnifications under the same conditions. Cells with five or more GFP-LC3 vacuole dots (puncta) were considered autophagy-positive. An average percentage of puncta-positive cells from nine fields/culture (total cells > 500) was calculated and used for statistical analysis.

### Glutathione S-Transferase (GST)-Pull Down Assay

GST-pull down assay was performed as previously described (29). 293 cells were transfected with indicated plasmid at 1:1 for 2 d. Cell lysates were extracted with GST-lysis buffer and 400 μg of total soluble proteins from each sample were incubated with 30 μl of glutathione sepharose bead slurry (GE Healthcare Life Sciences, Piscat-away, USA) overnight at 4°C. After extensive washing, the immunoprecipitates were subjected to Western blotting analysis. Anti-GST, anti-N-GFP or anti-PD-L1 antibodies were used to probe corresponding proteins.

### Membrane and Cytosol Protein Detection

U251 cells were transfected with p-FU-Venus or p-FU-Venus-PD-L1 plasmid for 24–36 h. Membrane and cytosol proteins were extracted using Membrane and Cytosol Protein Extraction Kit (Beyotime Biotechnology, Shanghai, China) according to manufature's instructions. Membrane and cytosol proteins were then subjected to Western blotting with Na^+^/K^+^ ATPase α1 and β-tubulin as internal control of membrane and cytosol proteins respectively.

### Statistical Analysis

All experiments were repeated independently for at least three times. The values were expressed as means ± SEM. Unpaired Student's test was used to compare between two groups of *in vitro* experiments. Paired Student's test was used to compare between two groups of animal experiments. Comparisons among multiple groups were performed through one-way ANOVA with Student–Newman–Keuls post-test. *P* < 0.05 was considered statistically significant.

## Results

### PD-L1 Is Prominently Elevated in Invasive Frontier GBM Cells *in vivo*

**Figure 1:**
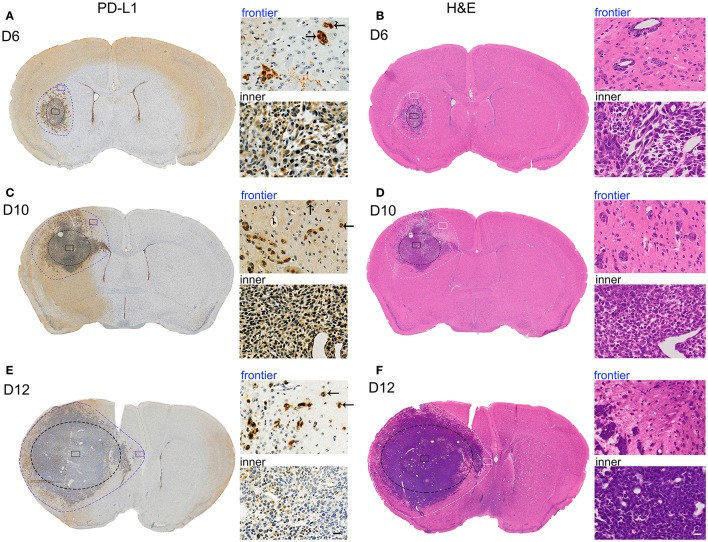
PD-L1 is preferentially elevated in invasive frontier GBM cells *in vivo*. Mouse G422 glioma cells (20,000 cells) were inoculated into right striatum of Kunming mice. 6, 10, and 12 days after initial cell implantation, the mice were perfused with PFA and paraffin-embedded (3 mice/group). Serial mouse brain slices were cut and the slices with maximal tumor-load were used for PD-L1 (3 slice/brain) or H&E staining (3 slice/brain). **(A)** Representative PD-L1 staining of glioma on day 6. Inner black-dot ellipse delineated the boundary of tumor parenchyma while the outer irregular dot circle delineated the infiltration boundary. Left panels showed the whole brain and right panels showed enlarged square images. Arrows indicated the invasive frontier glioma cells. **(B)** Representative H&E staining of glioma at day 6. **(C,E)** Representative PD-L1 staining of glioma at day 10 and 12. **(D,F)** Representative H&E staining of glioma at day 10 and 12.

To ascertain the relationships between intrinsic PD-L1 and glioma cell invasive behavior during GBM development, we examined PD-L1 expression in the whole brain at various stages of GBM development in a highly aggressive orthotopic mouse G422 model (Figure 1). PD-L1 as well as H&E staining clearly showed that the glioma tissue grew rapidly from day 6 to 12 (D6, D10, D12) after initial G422 cell-implantation. The tumor parenchyma in the left hemisphere was delineated with black-dot ellipse, and the tumor frontiers was delineated with blue (PD-L1, Figures 1A,C,E) or white-dot irregular circle (H&E, Figures 1B,D,F). Clearly, invasive GMB cells could migrate far away from its parenchyma mass at the early stage (D6) during GBM development. Along with the growth of glioma parenchyma, infiltration frontiers were also enlarged correspondingly. The frontier (blue) and inner (black) square micrographs in brain coronal sections were enlarged in right panels. It is clear that PD-L1 was prominently elevated in invasive G422 cells (indicated by arrows) in GBM infiltration frontier (Figures 1A,C,E). Such evidence establishes a strong positive correlation between PD-L1 and the aggressiveness of GBM cells.

### PD-L1-Altered Gene Expression Are Highly Enriched in Migration and PI3K/Akt-Actin Signaling

**Figure 2:**
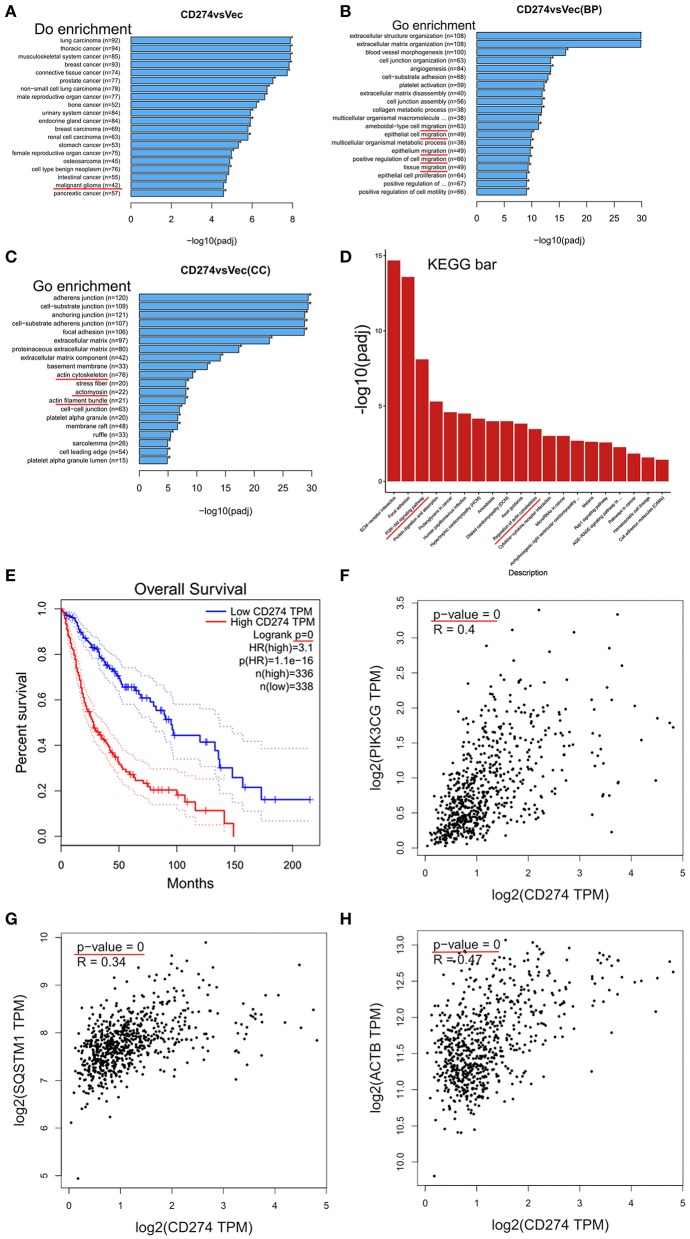
Transcriptomic analysis in PD-L1-overexpressed GBM cells. PD-L1 (CD274) was stably overexpressed in human U251 glioma cells. Total RNA was extracted from U251/PD-L1 or its control (U251/Vec) cells and subjected to mRNA-sequencing. Significant DEGs between U251/PD-L1 and U251/Vec groups with the following creteria: *P* < 0.005, Padj < 0.05 and absolute fold change ≥1.2 (*n* = 3). **(A)** DO (Disease Ontology) enrichment analysis results showed that 42 of significantly altered-genes upon PD-L1 overexpression were associated with malignant glioma (indicated by red line). **(B)** BP (biological process) from GO enrichment analysis results showed that PD-L1 overexpression was closely associated with various cell migration pathways (indicated by red lines). **(C)** CC (cellular component) from GO enrichment analysis results showed that PD-L1 overexpression was closely associated with several actin signaling pathways (indicated by red lines). **(D)** KEGG (Kyoto Encyclopedia of Genes and Genomes) pathway enrichment analysis results showed that PI3K-Akt and actin regulations are among the top altered pathways by PD-L1 overexpression (indicated by red lines). **(E)** Overall survival of glioma patients with PD-L1 expression from database http://gepia.cancer-pku.cn. Survival significant longer for patients with low PD-L1(*p* = 0). **(F–H)** Correlation analysis of PD-L1 with PIK3CG, SQSTM1 (p62), and ACTB (β-actin) showed that mRNA levels of PD-L1 were positively associated with those of PIK3CG, p62, and β-actin (*p* = 0) in glioma patient tissues (http://gepia.cancer-pku.cn).

To explore the underlying mechanisms of PD-L1^+^-GBM cells in the infiltration frontier, mRNA sequencing was conducted in PD-L1-overexpressed U251 glioma cells. Differential expressed genes (DEGs) between U251/PD-L1 and U251/Vec cells (i.e., CD274 vs. Vec) were selected by three criteria (i.e., *P* < 0.005, *P*adj < 0.05 and absolute fold change ≥1.2) and the selected DEGs were subjected to various systemic analysis. DO (Disease Ontology) enrichment showed that PD-L1-altered gene expression was significantly associated with malignant glioma (Figure 2A, indicated by red line). Gene ontology (GO) enrichment analysis showed that PD-L1-altered gene expression was largely associated with cell migration (BP: biological process, Figure 2B, indicated by red lines), actin-structure functions (CC: cellular component, Figure 2C, indicated by red lines), PI3K-Akt signaling pathway and Regulation of actin cytoskeleton (KEGG: Kyoto Encyclopedia of Genes and Genomes, Figure 2D, indicated by red lines). Further analysis of human glioma database (http://gepia.cancer-pku.cn) revealed that PD-L1 (CD274) was negatively associated with the prognosis of glioma patients (*p* = 0, Figure 2E). In addition, PD-L1 expression was significantly positively correlated with PI3K (*p* = 0, Figure 2F), SQSTM1 (p62, a negative marker of autophagic influx signaling) (32) (*p* = 0, Figure 2G) and ACTB (β-actin) (*p* = 0, Figure 2H) in human glioma tissues. The evidence together indicates that PD-L1 greatly affects glioma cell invasion and PI3K/Akt-actin signaling.

### PD-L1 Regulates Akt-p62-Autophagic Influx Signaling Upon Starvation

**Figure 3:**
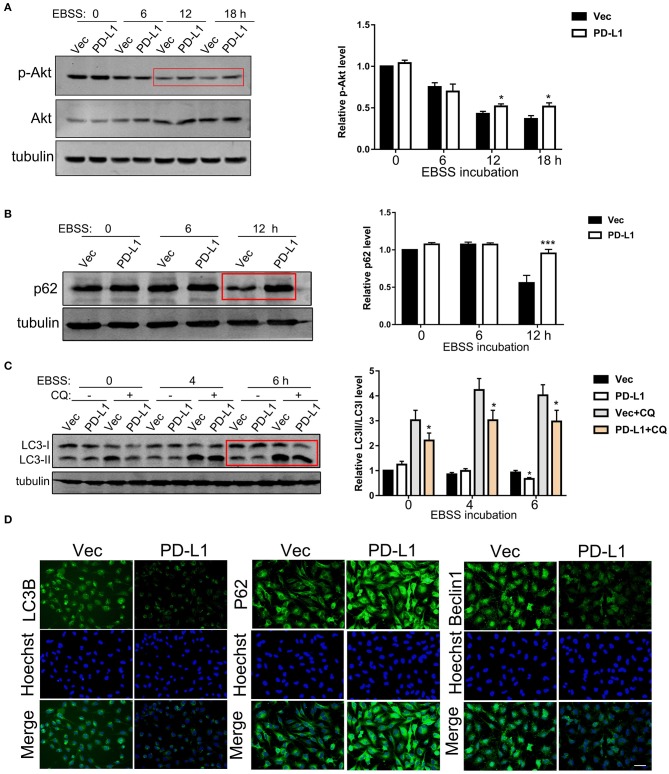
Effects of PD-L1 overexpression on Akt-p62-autophagic influx signaling upon starvation. **(A)** Western blot analysis of p-Akt, Akt and tubulin in U251/Vec and U251/PD-L1 cells subjected to various time of EBSS incubation. *P < 0.05 vs. corresponding Vec, *n* = 3. **(B)** Western blot analysis of p62 and tubulin in U251/Vec and U251/PD-L1 cells subjected to various time of EBSS incubation. ***P < 0.001 vs. corresponding Vec, *n* = 3. **(C)** Western blot analysis of LC3 and tubulin in U251/Vec and U251/PD-L1 cells subjected to various time of EBSS incubation with or without Chloroquine (CQ, 50 μM). ***P < 0.001 vs. corresponding Vec, *n* = 3. **(D)** Representative fluorescent immunostaining of LC3B, p62, and Beclin-1 in U251/Vec and U251/PD-L1 cells at EBSS-6 h. Bar, 20 μm. All experiments were repeated three times independently.

It is well known that GBM tissues suffer severe ischemia that causes necrosis and promotes GBM cell aggressiveness (1–3), suggesting that energy-deprivation stress is a major driving force for glioma cell invasion. Since PI3K/Akt signaling is pivotal in promoting cell survival and suppressing autophagy (6, 33), we further investigated the role of PD-L1 in regulating Akt-autophagic influx signaling in glioma cells with a EBSS-induced starvation model. Western blot results showed that PD-L1 overexpression did not evidently alter p-Akt/mTOR/p70S6K signaling under normal culture conditions (Figure 3A, EBSS-0 h and Figure S1). Upon EBSS incubation, p-Akt and p62 (a negative maker for autophagy influx) was prominently reduced in U251/Vec cells (Figures 3A,B), while LC3II was evidently increased (–CQ2, Figure 3C) as detected by Western blot. Cells with PD-L1 overexpression significantly reversed EBSS-induced p-Akt reduction, p62 reduction as well as LC3II elevation compared to their corresponding Vec controls (indicated by red boxes, Figures 3A–C). Consistently, fluorescent immunostaining showed that PD-L1 evidently reduced LC3B and Beclin-1 while increased p62 in starved LN229 glioma cells at EBSS-6 h (Figure 3D).

**Figure 4:**
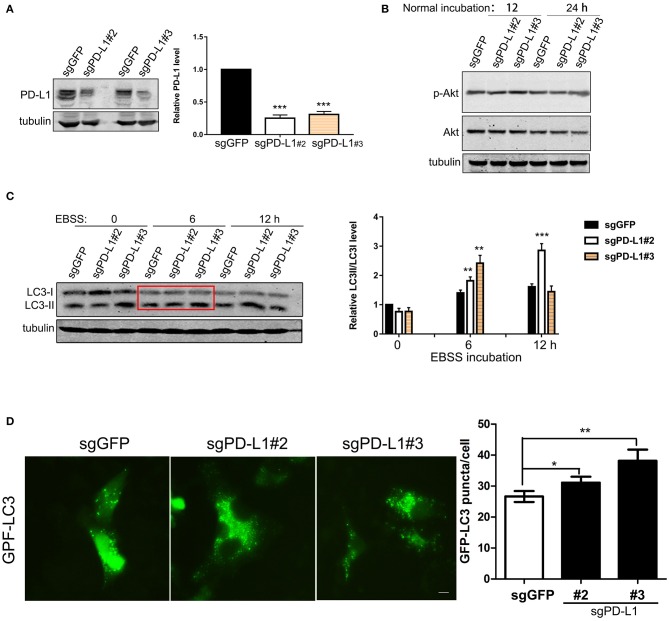
Effects of PD-L1 knockout on Akt, LC3 and autophagy puncta upon starvation. U251 cells were transfected with lentiviral sgGFP or PD-L1 and stable cell lines were selected. **(A)** Effects of PD-L1 knockout in U251/sgGFP, U251/sgPD-L1#2 and U251/sgPD-L1#3. ***P < 0.001 vs. sgGFP, *n* = 3. **(B)** Effects of PD-L1 knockout on p-Akt and Akt. Representative results showed that PD-L1 knockout did not alter p-Akt in U251 cells under normal incubation (*n* = 3). **(C)** Effects of PD-L1 knockout on LC3I and CL3II upon EBSS incubation. **P < 0.01 and ***P < 0.001 vs. corresponding sgGFP, *n* = 3. **(D)** Effects of PD-L1 knockout on LC3 puncta formation upon EBSS-12 h. 100–200 cells from each culture were counted. *P < 0.05 and **P < 0.01 vs. sgGFP, *n* = 3.

We further examined the effects of endogenous PD-L1 on autophagy influx signaling in glioma cells by knocking-out PD-L1 using CRISPR/Cas9 technique. Western blot results demonstrated that endogenous PD-L1 was prominently decreased in stable U251/sg-PD-L1 cell lines (single colnes #2 and #3, Figure 4A). Under normal culture conditions, PD-L1 knockout did not evidently affected p-Akt (Figure 4B) or p-mTOR (Figure S2) in U251 cells. Upon EBSS incubation (6 and 12 h), LC3II was significantly elevated in U251/sgPD-L1 cells as compared to its corresponding U251/sgGFP controls (Figure 4C). Consistently, fluorescent images of GPF-LC3-puncta clearly showed that the average number of GFP-LC3-puncta (representing autophagosome) per cell was significantly increased in U251/sgPD-L1 cells compared to its U251/sgGFP control at EBSS-12h (Figure 4D).

### PD-L1 128-237aa Fragment Interacts With Akt 112-480aa Fragment

**Figure 5:**
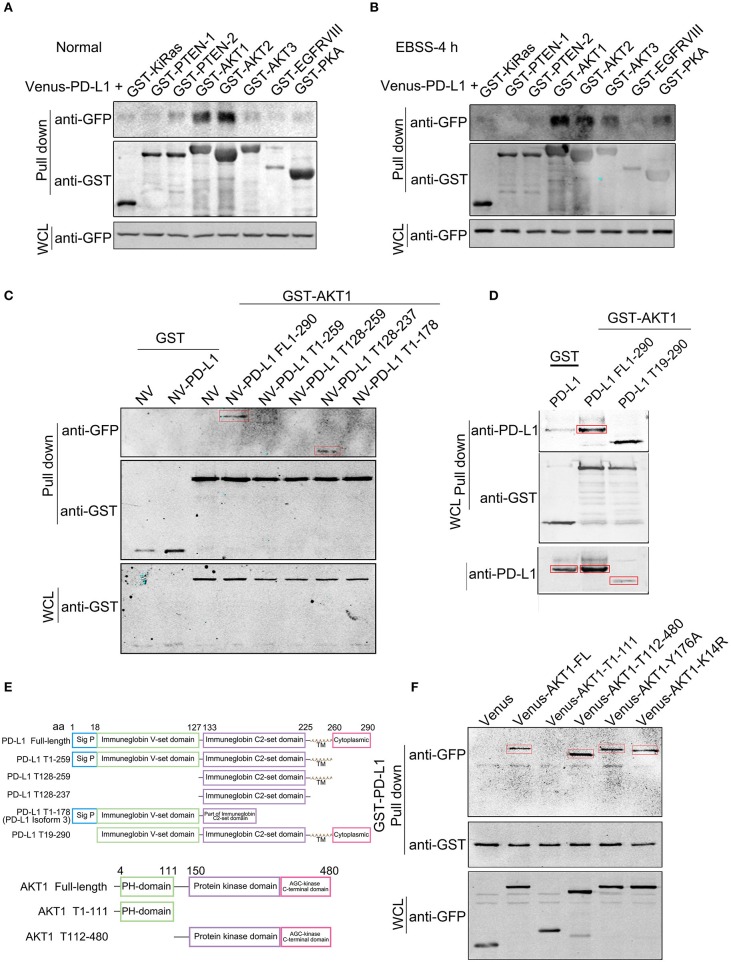
Identification of PD-L1-Akt binding domains. Venus (or N-terminal Venus)-tagged plasmids were co-transfected with GST-tagged plasmids in 293T cells. Equal amount of total soluble proteins were subjected to GST-pull down analysis. **(A,B)** Western blot analysis of the binding between Venus-PD-L1 and GST-KiRas, PTEN-1, PTEN-2, AKT1, AKT2, AKT3, EGFRVIII, and PKA under normal culture conditions **(A)** and EBSS-4 h **(B)**. WCL, whole cell lysate. **(C,D)** Western blot analysis of the binding between GST-AKT1 and NV-PD-L1 truncates. FL, full length; T, truncate. **(E)** Schematic diagram of PD-L1 and AKT truncates. **(F)** Western blot analysis of the binding between GST-PD-L1 and Venus-AKT truncates. All experiments were repeated at least three times independently.

In order to dissect the mechanism of PD-L1 action, we screened the binding partners of PD-L1 with various key upstream signaling proteins in PI3K/Akt pathway by GST-pull down experiment. The results clearly showed that PD-L1 preferentially bound to AKT1 and AKT2 compared to KiRAS, PTEN-1, PTEN-2, AKT3, EGFRVIII and PKA under normal culture conditions (Figure 5A) as well as EBSS-4h incubation (Figure 5B). Then, we further dissected the exact binding domain of PD-L1 for its AKT binding. We made a series of PD-L1 (full length 290 aa, FL) truncates by deleting 260–290 aa (PD-L1 T1-259), 179-290 aa (PD-L1 T1-178) from its C-terminal and 1-127 aa (PD-L1 T128-259), 1-127/238-290 (PD-L1 T128-259) (Figure 5C), 1-18 aa (PD-L1 T19-259) (Figure 5D) based on PD-L1 structure (upper panel, Figure 5E). GST-pull down results showed that PD-L1 FL1-290, T19-290, and T128-237 were the major truncates binding to AKT1 (indicated by red rectangles, Figures 5B,C). These results identified that PD-L1 128-237 fragment was required for its Akt binding.

We then seeked to identify the binding domain or key amino acid (aa) of Akt based on its structure (lower panel, Figure 5E). GST-pull down clearly showed that AKT1-FL (1-480 aa), T112-480 but not T1-111 bound to PD-L1 (Figure 5F). Single amino acid mutant of AKT Y176A (loss of TNK2 binding and membrane localization) (34) and K14R (substantial reduction of ubiquitination and loss of PIP3 binding) (35) did not evidently affect its interaction with PD-L1 (Figure 5F), suggesting that PD-L1/Akt interaction occurred in the cytosol via independent AKT K14 and Y176 sites.

### PD-L1 Facilitates Akt Membrane-Translocation and F-Actin Formation in Starved Glioma Cells

**Figure 6:**
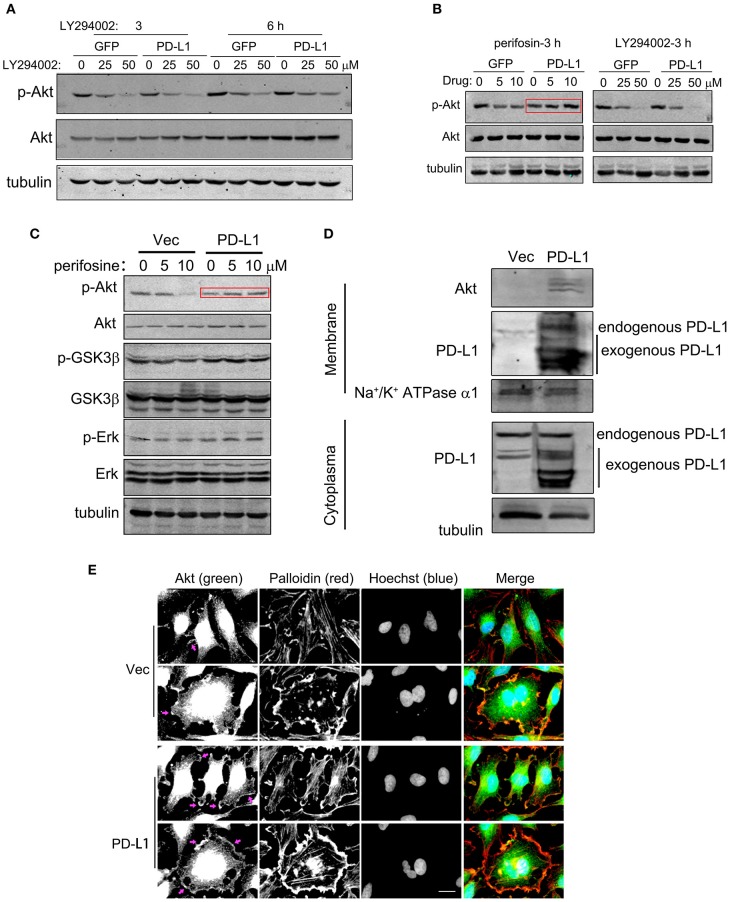
PD-L1 facilitates membrane-translocation of Akt. **(A)** Inhibitory effects of LY294002 on Akt activation. U251/GFP and U251/PD-L1 cells were treated with PI3K inhibitor LY294002 and subjected to Western blot analysis. **(B)** Differential inhibitory effects of Perifosin (inhibiting Akt PH domain function) and LY294002 on Akt activation in U251/GFP and U251/PD-L1 cells. **(C)** Effects of Perifosin-3 h on pAkt/Akt, p-GSK3β/GSK3β, and p-Erk/Erk in U251/PD-L1 cells. **(D)** Effect of PD-L1 on membrane Akt. Total proteins from cytoplasm and membranes of U251/PD-L1 or U251/Vec cells were isolated and equal amount of proteins were subjected to Western blot analysis. **(E)** Representative fluorescent microscopy showed membrane distribution of Akt and its co-localization with F-actin. Bar, 20 μm. All experiments were repeated three times independently.

Since PD-L1 selectively interacted with Akt 112–480 fragment but not its PH domain (1–111 aa) (Figure 5F), it is interesting to further figure out how PD-L1 promoted Akt activation upon starvation (Figure 3A). Full activation of Akt depends on Akt translocation from cytoplasm to cell membrane (via its PH domain) and PIP3/PDK action on cell membrane (36, 37), which can be pharmacologically inhibited by specific inhibitor Perifosin (37) and LY294002, respectively. Administration of LY294002 (GFP, Figure 6A) and Perifosine (GFP, Figure 6B; Vec, Figure 6C) evidently reduced p-Akt in U251/GFP cells. In PD-L1-overexpressed U251 cells, however, LY294002 but not Perifosin could reduce p-Akt level (PD-L1, Figures 6A–C). Such evidence suggests that PD-L1 protects the membrane-translocation of Akt. Western blot results demonstrated that membrane-bound Akt was evidently increased upon PD-L1 overexpression compared to Vec control (Figure 6D). Consistently, fluorescent immunostaining of Akt showed that membranous Akt (indicated by arrows) was more evident in PD-L1-overexpressed U251 cells, which was well co-localized with F-actin (Palloidin staining) (Figure 6E).

**Figure 7:**
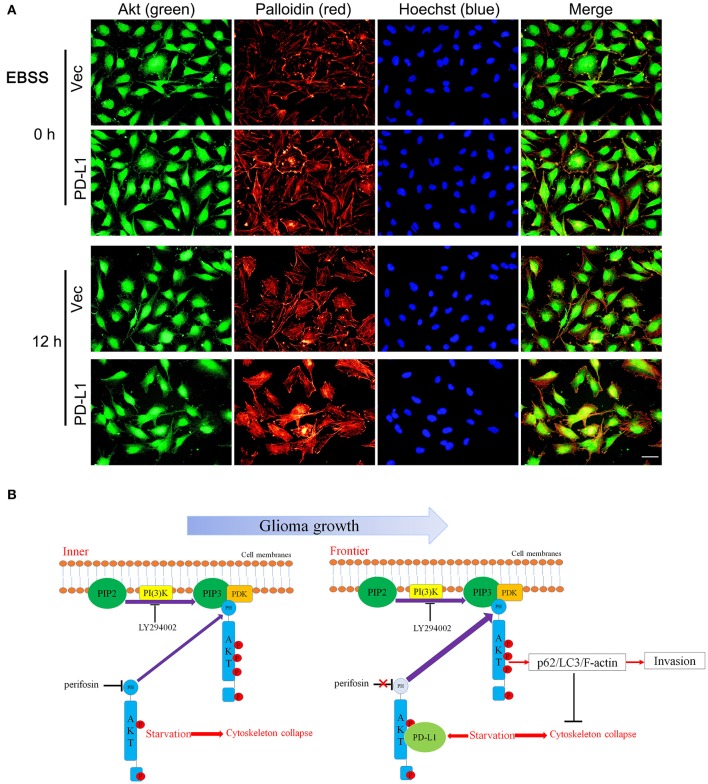
PD-L1 facilitates F-actin formation in glioma cells upon starvation. **(A)** Representative fluorescent microscopy showed elevated F-actin in U251/PD-L1 cells upon EBBS-12 h incubation. Bar, 20 μm. The experiments were repeated at least three times independently. **(B)** Proposed mechanism of PD-L1 action in invasive frontier glioma cells.

Finally, we examined the effects of PD-L1 on F-actin structure in starved glioma cells. Fluorescent images of Palloidin staining clearly showed that F-actin formation was evidently increased in PD-L1-overexpressed U251 cells under normal culture conditions as well as EBBS-12 h incubation (Figure 7A). Taken together, the evidence suggested that PD-L1 facilitated Akt translocation to cell membrane and Akt activation to modify cellular morphology via F-actin cytoskeleton during energy-deprivation stress.

## Discussion

In our present study, we discovered that PD-L1 elevation occurred predominantly in highly aggressive glioma cells. RNA sequencing revealed that high levels of PD-L1 in glioma cells mainly mediated cell migration and PI3K/Akt/actin signaling. PD-L1 reduced starvation-induced Akt inhibition, autophagic influx and F-actin collapse in glioma cells. Mechanistically, PD-L1 directly interacted with Akt, and this process involved PD-L1 128-237aa and Akt 112-480aa. The binding of PD-L1 to Akt facilitated membrane-translocation of Akt and thus elicited downstream biological effects in frontier glioma cells. These findings reveal a novel invasive mechanism in GBM.

PD-L1 is a pivotal negative regulatory molecule at the immune checkpoint axis and has complicated biological functions besides immune regulation (38). Detection of PD-L1 level in cancer cells is of primary importance for predicting its biological functions as well as prognosis of immune therapy in various cancers such as melanoma. Previous studies have reported that PD-L1 is widely expressed in most glioma cell lines including U251 (12, 29) and human glioma tissues (14), indicating important biological functions of PD-L1 in glioma. Here, we clearly demonstrated that PD-L1^+^-glioma cells were highly aggressive (Figure 1). Compared to rat/C6 and nude mouse/U87 glioma models, in which 1,000,000 or 250,000 glioma cells were implanted in the striatum and only a few glioma cells migrated outside from the tumor rim at day 14 or 24 later (29), in our orthotopic glioma model, only 20,000 G422 cells were microinjected into the striatum of mouse brain. In very short time (6 d) after initial glioma cell injection, PD-L1^+^-glioma cells had already migrated far from the glioma center (Figures 1A,B). Afterwards, invasive PD-L1^+^-glioma cells quickly reached the outer surface of cerebral cortex at day 10 (Figures 1C,D) and middle line of two hemispheres at day 12 (Figures 1E,F) after the initial glioma cell implantation. Obviously, the G422 glioma model mimics well the invasive pathological process of GBM. Since PD-L1 in cell membrane (39), exosome (40), and cytoplasm (29) are all pivotal for cancer development and progress, we can infer that the elevation of PD-L1 levels in those infiltrating glioma cells heavily contribute to their invasiveness.

To systematically reveal the role of PD-L1 in glioma cells, we conducted RNA sequencing in PD-L1-overexpressed human glioma U251 cells, which might mimic the status of higher PD-L1 levels in those infiltrating glioma cells. Bioinformatics analysis of DEGs clearly pointed out that increased PD-L1 in glioma was closely associated with glioma malignancy (Figure 2A), as well as distinct signaling pathways in invasive malignant glioma, such as cell migration, PI3K/Akt and actin organization (Figures 2B–D and Figures S1, S3). In a database of 674 glioma human samples, mRNA levels of PD-L1(i.e., CD274) were significantly positively correlated to those of PI3K (i.e., PI3KCG) and β-actin (i.e., ACTB) (Figures 2F,H), supporting our RNA-sequencing data. Also, bioinformatics analysis of glioma patient samples in opensource database showed that higher PD-L1 expression indicates poorer patient survival (Figure 2E), supporting that PD-L1's increase in infiltrating glioma cells is of clinical importance.

It is well known that GBM tissues suffer severe ischemic starvation, which is an important pathological factor driving aggressiveness (1, 2). PI3K/Akt is a pivotal signaling pathway promoting cell survival and suppressing autophagy under normal and ischemic conditions, while ischemic starvation is a major pathological stimulus for autophagy and actin cytoskeleton collapse (41, 42). It is conceivable that boundary GBM cells encounter energy-deprivation and higher PD-L1 expression may prevent GBM cell death and promote invasion via Akt-autophagy-actin signaling. Indeed, bioinformatics analysis of our RNA-sequencing data showed that PD-L1 affected autophagic signaling (Figure S2). By applying the glioma patient database, we found that mRNA levels of PD-L1 were significantly positively correlated to those of p62 (i.e., SQSTM1) (Figure 2G). Consistently, our experimental results demonstrated that PD-L1 significantly resumed EBSS starvation-induced Akt-autophagy signaling in glioma cells *in vitro* (Figure 3) but had minor effects under normal culture conditions (Figure 4B and Figure S4). The effects of PD-L1 overexpression on Akt increase and LC3 decrease were verified *in vivo* in a orthotopic rat/C6 glioma model (Figure S5). Further, via double-fluorescent immunostaining, we verified that higher PD-L1 levels in individual GBM cells were associated with lower Beclin1 and LC3 levels in GBM patient tissues (Figure S6). Such evidence consistently supported that PD-L1 suppressed autophagy via Akt induction/activation. In addition, PD-L1 increased F-actin formation and Akt/F-actin co-localization beneath cell membrane in glioma cells upon starvation (Figures 6E, 7A). In other cancer cells such as sarcoma cells, melanoma and ovarian cancer cells, PD-L1 also regulates Akt-mediated autophagic signaling (27, 31). This data largely supported our proposed PD-L1-Akt-p62-autophagy-actin-invasion mechanism in frontier GBM cells. Further collection of evidence is helpful to elucidate the exact invasive mechanism of GBM *in vivo*.

Importantly, we identified a novel mechanism by which PD-L1 regulate PI3K/Akt signaling. Among various upstream signaling proteins in PI3K/Akt pathway, we found that PD-L1 preferentially bound to Akt isoforms, and this became more evident upon EBSS incubation (Figure 5 and Figure S7). Moreover, we dissected the binding element of PD-L1 and Akt. Our data revealed that the 128-237aa fragment of PD-L1 and the 112-480aa fragment of Akt were responsible for PD-L1/Akt interaction (Figure 5). Mutation of K14 and Y176, two active sites for the membrane-translocation of Akt, did not alter PD-L1/Akt interactions (Figure 5F), suggesting that PD-L1/Akt interaction occurred mostly in the cytoplasm. Upon PD-L1 overexpression, membrane-bound Akt was prominently increased (Figure 6D), supporting that cytoplasmic PD-L1/Akt interactions facilitated Akt translocation from cytoplasm to cell membrane, which is required for full activation of Akt (36). Interestingly, LY294002 (inhibiting p-Akt via PI3K) but not Perifosin (inhibitor of membrane-translocation of Akt and p-Akt via PH domain) (37) could abolished Akt phosphorylation in the presence of PD-L1 overexpression (Figures 6A–C). Such evidence suggested that the binding of PD-L1 to non-PH domain of Akt may prevented Perifosin action via some unknown mechanism, thus strengthened the attachment of Akt PH-domain to cell membrane and facilitated its full phosphorylation (Figure 7B). Further study on the mechanisms of PD-L1/Akt interactions requires more experimental data in the future.

In summary, our findings indicate the following model for PD-L1 actions in GBM (Figure 7B): Along with the rapid GBM growth, inner GBM cells suffer severe starvation, which causes cytoskeleton collase; while frontier GBM cells suffer less severe starvation, which induces PD-L1 expression and its binding to Akt non-PH domain. This reinforces Akt's membrane-binding and leads to increased full activation of Akt as well as its downstream p62/LC3/F-actin signaling, preventing starvation-induced Akt inhibition and facilitating GBM cell invasion. By combining our previous findings that PD-L1 promotes EMT of glioma cells, we speculate that PD-L1/Akt/autophagy/F-actin is a key driving force for GBM aggressiveness, which serves as a potential therapeutic target for GBM.

## Data Availability Statement

The datasets generated for this study can be found in the NCBI (https://www.ncbi.nlm.nih.gov/geo/query/acc.cgi?acc=GSE107581).

## Ethics Statement

The animal study was reviewed and approved by Experimental Animal Ethics Committee, Tongji Medical College, Huazhong University of Science and Technology.

## Author Contributions

RC, XX, XQ, FL, CL, YL, XL, and GJ performed the experiment. RC, FP, XQ, and XC data analysis, wrote the manuscript, and contributed to discussion. FH and DL contributed to discussion. RC, FP, XQ, and XC designed and wrote the manuscript.

### Conflict of Interest

The authors declare that the research was conducted in the absence of any commercial or financial relationships that could be construed as a potential conflict of interest.
