# Supplementary material for: The Binding of PD-L1 and Akt Facilitates Glioma Cell Invasion Upon Starvation via Akt/Autophagy/F-Actin Signaling
Source: Front Oncol. 2019 Dec 3;9:1347. doi: 10.3389/fonc.2019.01347 (PMC6901431; doi:10.3389/fonc.2019.01347)
Supplement: Supplementary file 1 [file Data_Sheet_1.PDF]

Title page

The Binding of PD-L1 and Akt Facilitates Glioma Cell Invasion upon Starvation via Akt/autophagy/F-actin Signaling

Ruo Qiao Chen[1,†], Xiao Hong Xu[2,†], Feng Liu[2], Chun Yang Li[2], Yuan Jun Li[2], Xiang Rui Li[1], Guo Yong Jiang[1], Feng Hu[3], Di Liu[4], Feng Pan[4*], Xin Yao Qiu[2*], Xiao Qian Chen[2*]

[1]School of Basic Medicine, Tongji Medical College, Huazhong University of Science and Technology, Wuhan, Hubei, China

[2]Department of Pathophysiology, School of Basic Medicine, Tongji Medical College, Huazhong University of Science and Technology, Wuhan, Hubei, China

[3]Department of Neurosurgery, Tongji hospital, Tongji medical college, Huazhong University of Science and Technology, Wuhan, Hubei, China

[4]Department of Urology, Union Hospital, Tongji Medical College, Huazhong University of Science and Technology, Wuhan, Hubei, China

[*]Correspondence: Feng Pan, panfeng@hust.edu.cn; Xin Yao Qiu, qiu.xinyao1991@outlook.com; Xiao Qian Chen, chenxq@mails.tjmu.edu.cn

[†]These authors have contributed equally to this work

Conflicts of Interest Statement: The authors declare no conflicts of interest.

Running title: PD-L1/Akt interaction promotes GBM invasion

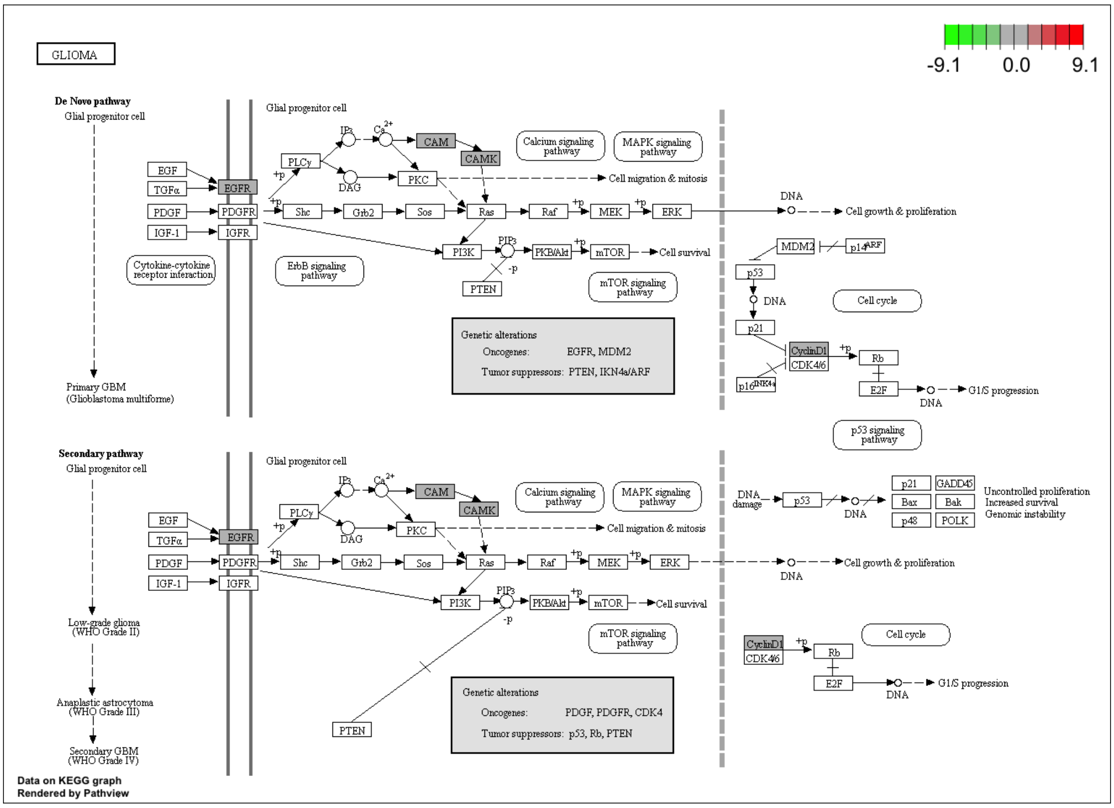

Figure S1. KEGG pathway analysis of PD-L1-altered genes in human glioma.

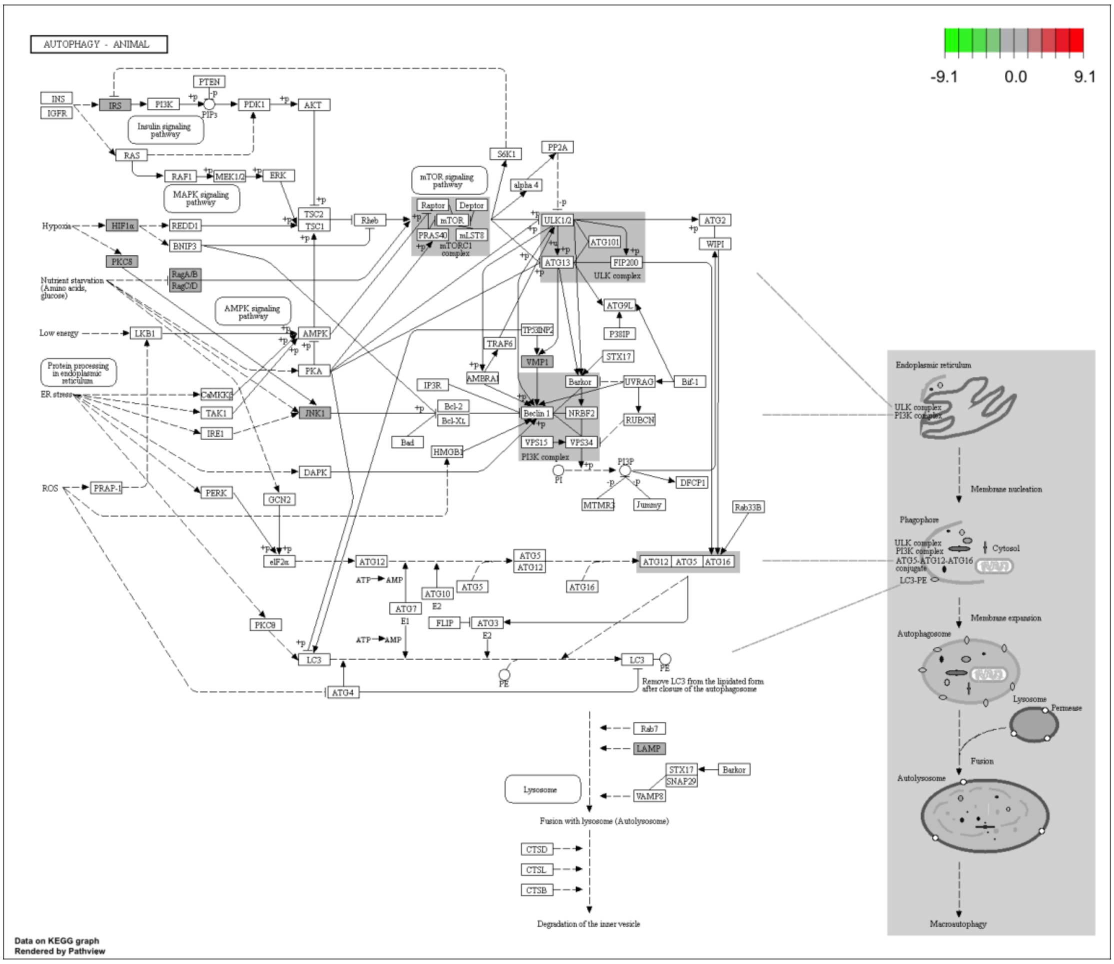

Figure S2. KEGG pathway analysis of PD-L1-altered genes in autophagy process.

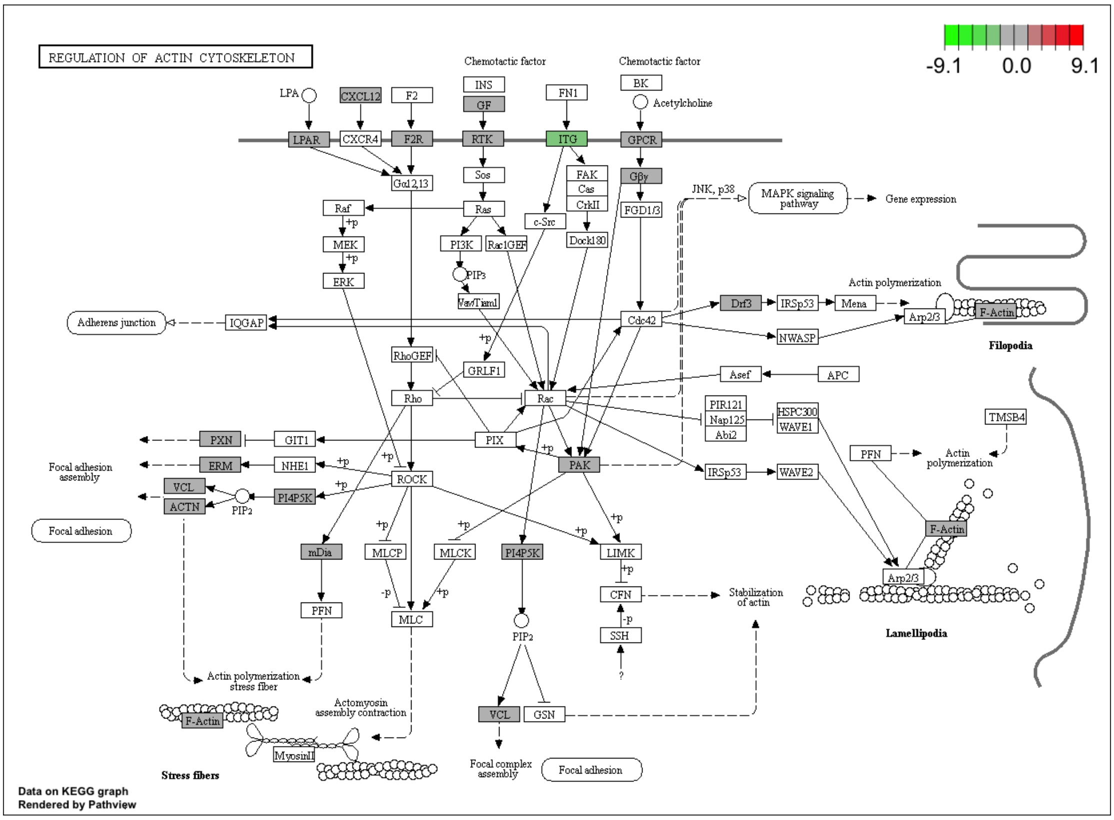

Figure S3. KEGG pathway analysis of PD-L1-altered genes in regulation of actin cytoskeleton.

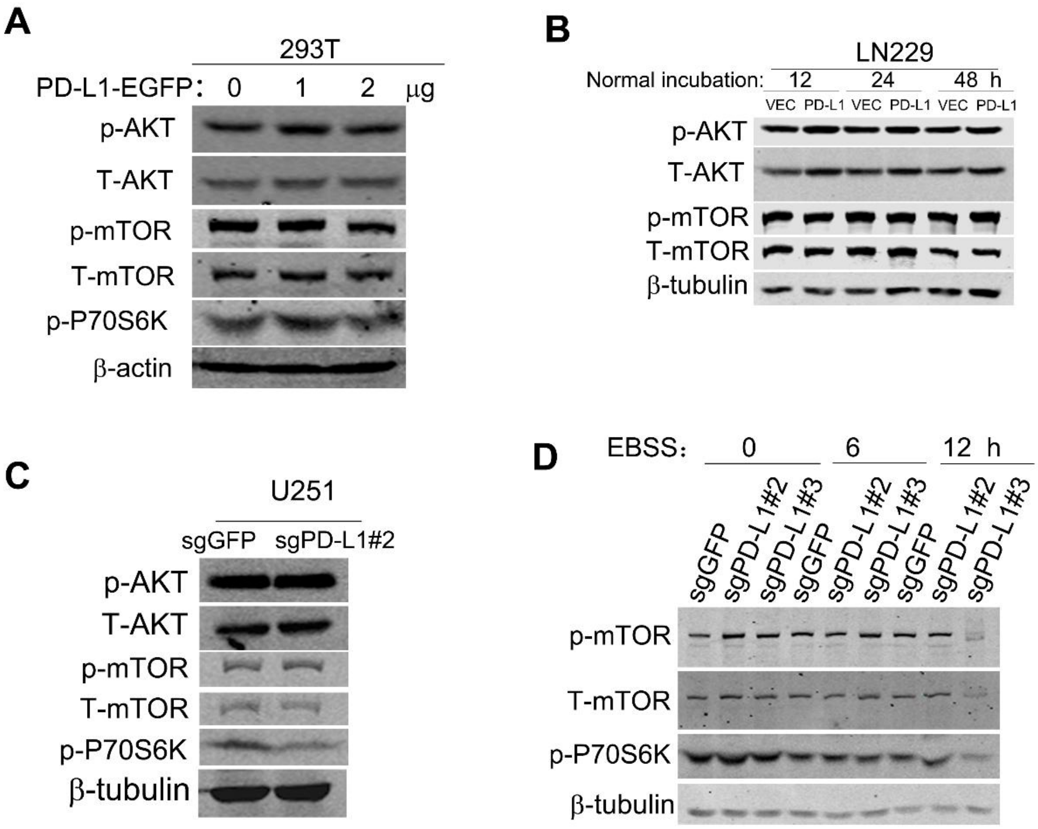

Figure S4. Effects of PD-L1 on Akt/mTOR signaling. (A) Effects of p-PD-L1-EGFP plasmid transfection of different plasmid amounts on p-Akt, Akt, p-mTOR, mTOR, p-P70S6K expression in 29T cells. (B) Effects of PD-L1 overexpression on p-Akt, Akt, p-mTOR, mTOR expression in LN229 cells at various time points after initial cell seeding. (C) Effects of PD-L1 knockout (sgCD274#2) on p-Akt, Akt, p-mTOR, mTOR, p-P70S6K expression in U251 cells. (D) Effects of PD-L1 knockout (sgCD274#2, #3) on p-mTOR, mTOR, p-P70S6K expression in U251 cells upon EBSS incubation.

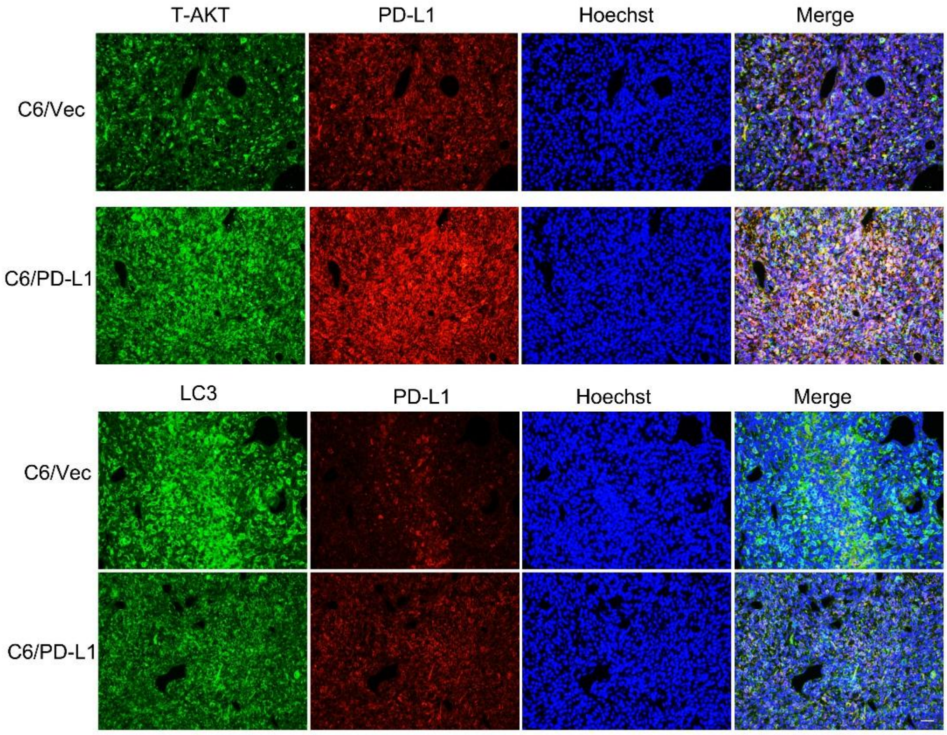

Figure S5. Double fluorescent-immunostaining shows the effects of PD-L1 overexpression on Akt and LC3 expression in orthotopic rat/C6 glioma tissues. C6 cells were lentiviral-infected for PD-L1 overexpression (C6/PD-L1). Equal amounts of C6/PD-L1 or C6/Vec were microinjected into right striatum of rats and the glioma tissues were subjected to analysis at 20 d after initial C6 cells injection. Bar, 20 μm.

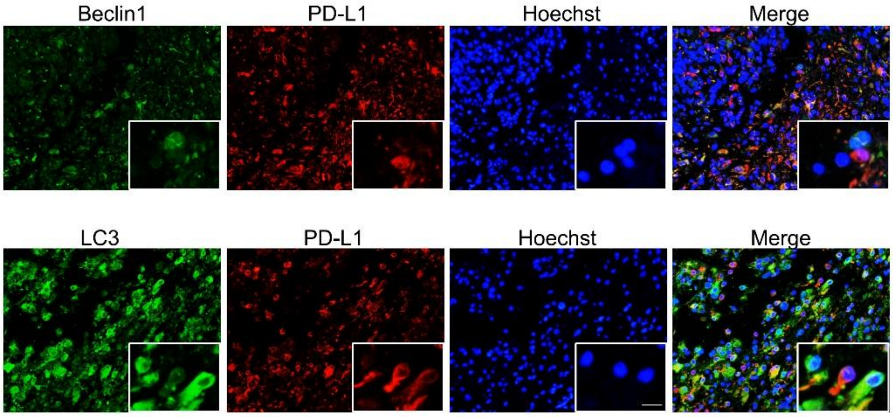

Figure S6. Double fluorescent immunostaining shows that higher PD-L1 expression in individual GBM cells is associated with lower Beclin-1 and LC3 expression in GBM patient tissue. Bar, 20 μm.

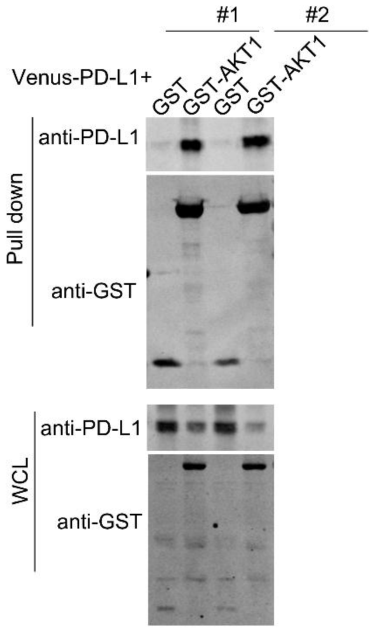

Figure S7. GST-pull down shows that PD-L1 binds to Akt. WCl, whole cell lysate.
